# Increase of Brivaracetam serum concentration with introduction of Cenobamate

**DOI:** 10.3389/fphar.2025.1571376

**Published:** 2025-04-01

**Authors:** Lena Bender, Martin Hirsch, Andreas Schulze-Bonhage

**Affiliations:** Epilepsy Center, University Medical Center, University of Freiburg, Freiburg, Germany

**Keywords:** antiseizure medication, epilepsy, brivaracetam, cenobamate, interaction, hepatic enzyme inhibition, serum levels

## Abstract

**Introduction:**

Cenobamate is a new antiseizure medication approved for polytherapy of focal epilepsy with complex hepatic metabolism and effects on liver enzymes. So far, data are limited with regard to possible interactions with other antiseizure medications. We here report effects of Cenobamate on serum levels of Brivaracetam, a SV2-agent modulating presynaptic neurotransmitter release.

**Methods:**

Retrospective analysis of Brivaracetam serum concentrations with new introduction of Cenobamate with Brivaracetam as a constant baseline antiseizure medication in 19 patients with focal epilepsy. Statistical analysis using paired Fisher´s exact t-Test.

**Results:**

New introduction of Cenobamate lead to a statistically significant increase in Brivaracetam serum concentrations with a mean increase by 27%. This was infrequently accompanied by adverse effects.

**Discussion:**

New introduction of Cenobamate to a pre-existing antiseizure regimen containing Brivaracetam leads to considerably increases in Brivaracetam, probably related to inhibition of CYP2C19. This needs to be taken into account when interpreting changes in treatment efficacy, but also when relating potential adverse effects to baseline vs. newly introduced treatment.

## Introduction

Focal epilepsies account for 61% of epilepsies worldwide. Even though antiseizure treatment has been significantly optimized in recent years through the introduction of new antiseizure medication with extended and partially new mechanisms of action, up to 31% (Janmohamed et al., 2023) of all patients with focal epilepsy remain refractory to pharmacological treatment. Polytherapy, the use of new ASM and the understanding of its pharmacokinetics are necessary to achieve satisfactory results in the majority of patients (Operto et al., 2023). The resulting drug load may contribute to the burden of epilepsy in this difficult-to-treat population, and drug interactions may contribute to this (Operto et al., 2023).

Having been approved by the US Food and Drug Administration in November 2019 and by the European Medicines Agency in March 2021 for the adjunctive treatment of focal onset seizures in adults, Cenobamate (Ontozry, Angelini) plays a pivotal role in the treatment of difficult to treat and pharmacorefractory focal epilepsy. According to real life studies, Cenobamate (CNB) can achieve a >50% seizure reduction in 55%–63%, and a >75% reduction in 27,9%–35% (Novitskaya et al., 2024; Villanueva et al., 2023), while 13,3% (Villanueva et al., 2023) and 14% (Steinhoff et al., 2024) of patients with focal epilepsy obtain seizure freedom. Furthermore CNB contributes to a significant reduction of concomitant ASMs (Roberti et al., 2024). Assenza et al. even suggest the use of CNB as a therapeutic response biomarker as they provide evidence of qEEG modulations which correlate with CNB induced changes in seizure frequency (Assenza et al., 2025).

CNB is a tetrazole carbamate derivate. The favourable effects on seizure outcome are attributed to its dual complementary mechanism of action with blockade of voltage-gated sodium channels (VGSCs) (Keam, 2020; Löscher et al., 2020) and allosteric modulation of synaptic and extrasynaptic γ-aminobutyric acid type A receptors (GABA-A- receptors) on the other side.

The absorption rate after oral administration is almost 90% (Steinhoff et al., 2024). The maximum serum concentration (Cmax) is reached after 0.8–3.5 h and a plateau for 6–12 h after administration. The elimination half-life time (t ½) diverges between 30 and 76 h (Vernillet et al., 2020). The steady-state serum concentration is reached after 2 weeks of daily dosing.

CNB is metabolized to a major amount in the liver through glucuronidation (via UGT2B7 and UGT2B4) and oxidation (via CYP2E1, CYP2A6, CYP2B6), and to a lesser extent through hydroxilation (via CYP2C19 and CYP3A4/5) (Vernillet et al., 2020). In the first 24 h the parent drug is the predominant circulating moiety.

The concentration-time profile shows a non-linear multiphase elimination. with the parent drug and its metabolites being eliminated by a distinct mechanism. The main route of excretion is renally (>80%). CNB is a CYP2C19-inhibitor, and a CYP2C8- and CYP2B6-inductor. Furthermore it exhibits a dose-dependent induction of CYP3A4-activity and has a minor effect on CYP2C9 activity (Greene et al., 2022). By inhibiting CYP2C19 CNB leads to an increase of the serum level of PHY and PB (84% and 37%) (Roberti et al., 2021) while its own AUC diminishes up to 28% vs. 15%. Likewise an increase of the active metabolite of clobazam with concomitant CNB therapy is observed due to CYP2C19-inhibition. A dose reduction is to be considered. In contrast, the blood level of Lamotrigine is reduced by 21%–52% (Roberti et al., 2021). Roberti et al. (2021) postulated potential interactions between CNB and Brivaracetam (BRV) in an increase of the BRV serum level through CYP2C19-inhibition with a consecutive need of dose-adaption of BRV. BRV is thought to reduce the release of neurotransmitters by serving as a ligand to synaptic glycoprotein SV2A in the brain with a 15–30 times higher selective affinity than its analogue Levetiracetam. This may contribute to a more favourable profile of side effects with comparable antiseizure effect to Levetiracetam (Khaleghi and Nemec, 2017). Hirsch et al. (2018) could demonstrate that in 57.1% of patients that switched from Levetiracetam to BRV because of affective side effects a better tolerability was achieved.

BRV is completely absorbed after oral administration and has a linear pharmacokinetic profile. The plasma protein binding is low. It underlies a high amount of biotransformation processes through different metabolic pathways and over 90% are eliminated renally (Laura et al., 2008).

The three main metabolites are formed by amidase-mediated hydrolysis of the acetamide group (60%), CYP-mediated hydroxylation on the propyl side chain (30%) (Dean et al., 2012), and a combination of the two. The metabolites are not pharmacologically active. *In vitro* inhibition assays and the result of a gemfibrozil clinical trial demonstrated that CYP2C8 and CYP2C9 were not involved, but CYP2C19 is predominantly mediating the hydroxylation pathway (Molteni et al., 2024).

This results in a significant decrease of the hydroxyl metabolite (2–10 fold) in people with genetic CYP2C19-variations, in whom BRV blood level can increase between 22% and 42% depending on the genetic variant (Dean et al., 2012), or patients on a therapeutic regime with CYP2C19-inhibitors. Compensatory dose adaptations have been discussed to compensate for this effect.

We here, for the first time provide data of the pharmacokinetic interactions resulting from Co-administration of the CYP2C19-inhibitor CNB to BRV as a CYP2C19-substrate, and possible clinical correlates thereof.

## Methods

Between June 2021 and October 2023 112 adult patients with refractory focal epilepsy were started on CNB as adjunctive treatment as part of a real-world, long-term, prospective, open-label trial (Novitskaya et al., 2024) at the tertiary epilepsy centre at the university clinic in Freiburg. For the analysis of potential pharmacokinetic effects of CNB on BRV, we included all patients fulfilling the following criteria: unchanged baseline medication including BRV, at least 2 determinations of serum concentrations of BRV at the time of introduction and with uptitrated CNB to at least 100 mg/day, available documentation of dosages of CNB and BRV, and a stable BRV dosage through time of follow up.

Our aim was to compare the changes in serum concentrations of BRV in the initial phase of CNB-titration (group 1) with the concentrations in the further course of treatment (group 2).

Group 1 consisted of 19 patients (group 1, n = 19) in the initial titration phase, with a CNB dosage of 0–12.5 mg/day at first assessment (baseline dosage, t1) and re-assessment after introduction of CNB to 100–350 mg/day (t2). Group 2 consisted of 11 patients (group 2, n = 11) who were already under a stable CNB dose of 50–200 mg/d at first assessment (t3) and in whom the CNB dosage was further increased until the second assessment (t4) (Table 1.; Table 2.; Table 3). Patients with dose adaptions of BRV in the titration phase of CNB or in the further course of treatment were excluded from the analysis. Adverse events were noted. In case of occurrence the correlation with the BRV dosage, BRV concentration, and CNB dosage was investigated (Table 4). CNB concentrations were not tested.

**TABLE 1:** Patient demographics and baseline clinical characteristics.

Characteristics	Titration of CNB (group 1)		Change of CNB during treatment (group 2)	
Patients, n	19		11	
Age, years, mean, range	42, 21–69		40, 23–60	
Gender, female, male	9, 10		6, 5	
Age at epilepsy onset, years, mean, range	18, 1–47		10, 1–20	
Duration of epilepsy, years, mean, range	24, 5–53		30, 12–53	
Number of ASMs, mean, range	2,53, 2–3		2,63, 2–3	
CNB dosage at t1/t2/t3/t4, mg, mean, range	0	171,1, 100–350	136,4, 50–250	213,6, 100–250
BRV dosage, mg, mean, range	205,3, 100–300		213,6, 50–300	
BRV serum level at t1/t2/t3/t4, µg/L, mean, range	1562,3, 9.2–2,766	1963,5, 890–3,700	2144,7, 420–4,321	1891,5, 489–4,044

**TABLE 2:** Single subject description of BRV and CNB dosage, and BRV serum level, group 1.

Group 1	BRV dosage at t1, mg	CNB dosage at t1, mg	BRV serum level at t1, µg/L	CNB dosage at t2,mg	BRV serum level at t2, µg/L
p1	200	12,5	511	200	1,566
p2	300	12,5	2073	250	2,964
p3	200	12,5	2,766	150	3,700
p4	300	12,5	1982	150	2054
p5	200	12,5	1,510	100	1931
p6	150	12,5	1,518	150	2,274
p7	200	12,5	2,545	150	2,116
p8	100	12,5	950	100	1,299
p9	200	12,5	1723	300	1842
p10	200	12,5	2,206	150	3,024
p11	250	12,5	2004	350	1,580
p12	250	12,5	641	200	890
p13	300	12,5	2,269	100	2,346
p14	150	12,5	824	100	917
p15	100	12,5	1,070	200	1,356
p16	250	12,5	1,247	200	1,605
p17	150	12,5	9,2	100	2,556
p18	150	12,5	1,298	150	1,361
p19	250	12,5	2,537	150	1925

**TABLE 3:** Single subject description of BRV and CNB dosage, and BRV serum level, group 2.

Group 2	BRV dosage at t1, mg	CNB dosage at t3, mg	BRV serum level at t3, µg/L	CNB dosage at t4, mg	BRV serum level at t4, µg/L
p1	200	150	2005	250	1,521
p2	300	150	2054	350	2,587
p3	300	150	2,572	250	1,545
p4	200	50	1,545	100	1,492
p5	250	100	4,321	150	4,044
p6	300	100	2,346	150	2,005
p7	200	200	2,487	250	1,148
p8	50	200	420	250	489
p9	150	100	2,556	150	2,072
p0	150	150	1,361	250	852
p11	250	150	1925	200	3,051

**TABLE 4:** Patients with minor adverse events: demographics, BRV and CNB dosages, and BRV serum level.

	Patient 1	Patient 2	Patient 3
Age	19	31	27
Gender	female	male	female
Age at epilepsy onset, years	16	24	22
Duration of epilepsy, years	3	7	5
Life time ASM, n	5	4	11
CNB dosage at time of reported fatigue	150	300	250
BRV dosage at time of reported fatigue	100	200	100
BRV serum level at time of reported fatigue	240	1,172	1,143

Whereas more recent studies proved Ultra-High Performance Liquid Chromatography–Tandem Mass Spectrometry as a highly sensitive and selective method to quantify CNB in plasma (Molteni et al., 2024), in our study BRV serum concentration was maintained by LC-MS (Liquid Chromatography-Mass Spectrometry) according to the latest ICH Guideline M10 for Bioanalytical Method Validation. (Stockis et al., 2014; European Medicines Agency, 2025).

No genetic testing on CYP-characteristics was performed.

To investigate the effect of CNB dose changes on the BRV serum concentration a two-sided Fisher’s exact test was applied in R and Excel.

## Results

The study included 30 adult patients with refractory focal epilepsy, aged between 21 and 69 years (M = 41,26), 14 males and 16 females. There were no differences in gender or age distribution between group 1 (21–69 years, females 9, males 10), and group 2 (23–60 years, females 6, males 5).

**FIGURE 1:**
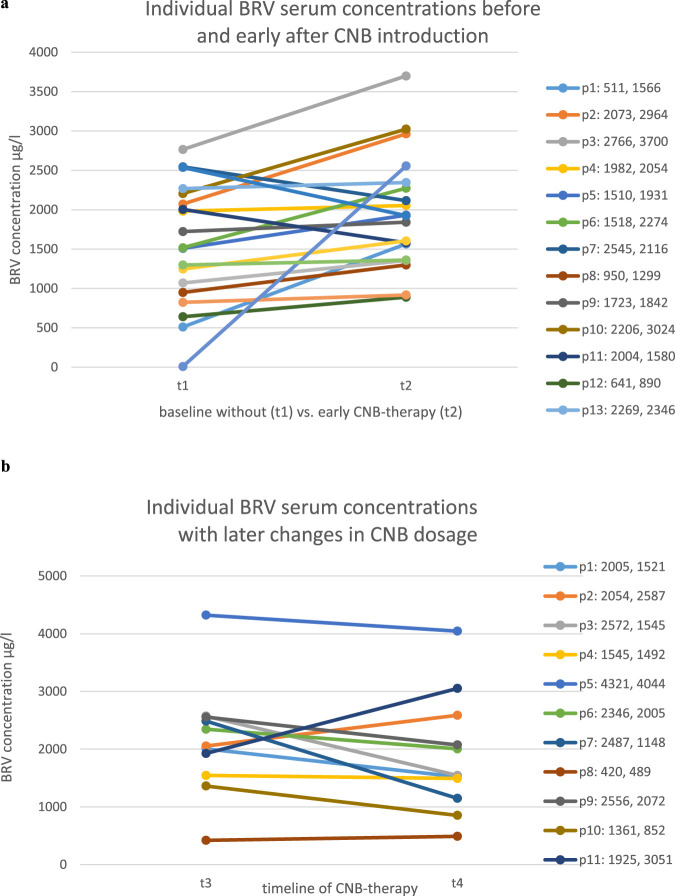
**(a)** Brivaracetam serum concentrations prior to CNB introduction (t1; CNB dosage 0–12.5 mg/day) vs. after introduction by at least 100 mg/day (t2) on a per-patient basis. **(b)** Brivaracetam serum concentrations with established CNB treatment (t1: CNB dosage >100 mg/day) later in the course of treatment (t2: 100–400 mg/day) on a per-patient basis.

**FIGURE 2:**
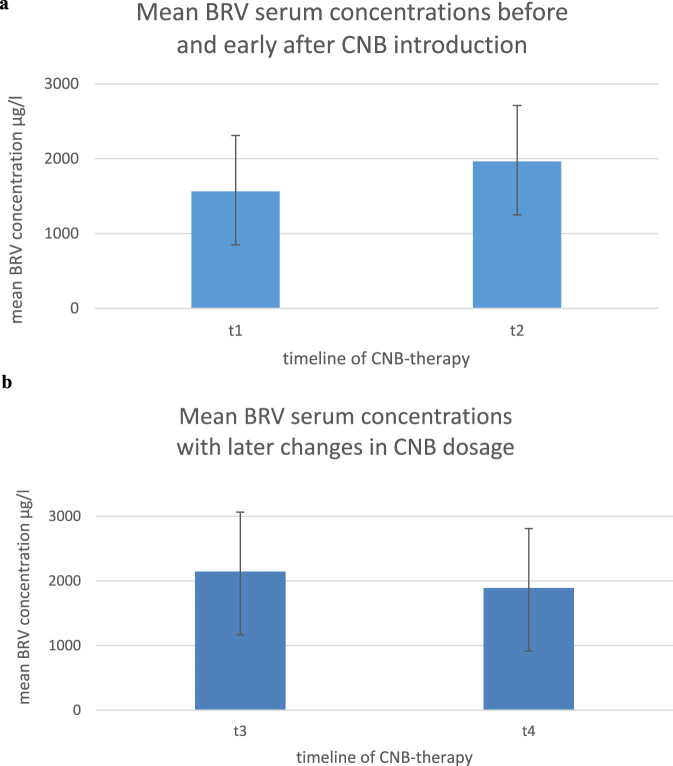
**(a)** Brivaracetam serum concentrations prior to CNB introduction (t1; CNB dosage 0–12.5 mg/day) vs. after introduction by at least 100 mg/day (t2) as means ± standard deviation. **(b)** Brivaracetam serum concentrations with established CNB treatment (t1: CNB dosage >100 mg/day) later in the course of treatment (t2: 100–400 mg/day) on a per-patient basis as means ± standard deviation.

In group 1 (n = 19) the results of the BRV serum concentration at CNB doses of 0/12,5 mg/d (t1) showed a mean of 1562,3 μg/L (±748,29 g/L SD); the mean BRV serum concentration after the initial titration (t2) was 1963,47 μg/L (±712,24 μg/L SD) as shown in Figure 2a. 16/19 patients (84,21%) patients showed an increase of BRV serum concentrations at stable daily dosages of BRV as demonstrated in Figure 1a. The results were highly significant (p < 0.005). On average the concentration increased by 27%.

In contrast, there were no significant changes in BRV concentrations after completed titration phase of CNB (t3, t4, >100–400 mg/d) as shown in Figure 1b. The results of the BRV serum concentration after completed titration (t3) showed a mean of 2144,73 μg/L (±918 μg/L SD) vs. 1891,45 μg/L (±978,13 μg/L SD) at a second time of comparison in the further course of CNB-treatment (t4) demonstrated in Figure 2b. 3/11 patients (27,3%) showed an increase of the BRV serum level while 8/11 (72,73%) did not (differences n. s.).

The two-sided Fisher’s exact test revealed a significant difference between the distribution of slopes (p < 0.005).

The overall tolerability of the combined therapy with BRV and CNB was good. There was no report of major adverse events associated with increasing drug level of BRV, however, three patients complained about a transient fatigue after increase of CNB-dosage with consecutive reduction of BRV dosage and clinical improvements thereafter. Only in one patient with constant BRV dosage, fatigue was accompanied by an increase in BRV serum concentration as shown in Table 2.

## Discussion

We here found a statistically significant, yet clinically mostly asymptomatic increase of BRV levels with new introduction of CNB to a pre-existing treatment with BRV. By inhibiting CYP2C19 CNB is known to increase the serum level of Phenytoin and Phenobarbital while its own serum concentration decreases when being part of a polytherapy regime that includes other enzyme-inducers. In addition, CNB has similar pharmacodynamic properties with certain ASM, thus co-administration is more likely to cause additive adverse effects, such as in combination with i.e., Lacosamide. A potential pharmacokinetic effect of CNB in BRV shown here had been postulated in a review of pharmacokinetic properties of CNB by Roberti et al. (2021), suggesting that an increase due to inhibition of CYP2C19 might necessitate a dose reduction of BRV. We could confirm the pharmacokinetic interaction, yet with minor pharmacodynamics consequences. This may well reflect the high therapeutic index of SV2A blockers (Schulze-Bonhage, 2011).

Whereas we found significant changes of BRV serum concentrations with new introduction of CNB, dose modifications in the further course of combined treatment were no longer significant. This suggests that relevant inhibition of CYP2C19 takes place already at lower dosages, e.g., in the range of 50–100 mg CNB/day, with minor effects of further increases in dosages. This resembles other inhibitory pharmacokinetic interactions, as e.g., seen with valproate and lamotrigine (May et al., 1996). Certainly this findings needs to be confirmed in larger patient populations.

Adverse effects were limited, however, with rare increases in fatigue reported. The finding that reductions in Brivaracetam dosage lead to the cessation of this adverse effects may point to both, pharmacodynamics and pharmacokinetic interactions of the two drugs. Similarly, in the few patients reporting daytime fatigue, reduction of BRV dosage was effective to ameliorate this symptom of combined administration of the two antiseizure medications and thus may not only be indicative of dose-related adverse effects of CNB administration.

The sample size of 30 patients and the retrospective design are limitations of this study. Thus larger prospective studies are needed to confirm the reported pharmacokinetic interaction. Furthermore a detailed CYP-assessment may provide additional information about the interactions between the two drugs across the spectrum of applied dosages.

## Conclusion

Our findings provide first evidence that in the initial titration phase of CNB, serum concentrations of co-administered BRV increase in the majority of patients, whereas there is no significant increase in the course of combined therapy with CNB and BRV with further up-titration of CNB. This reflects an expected effect on CYP enzymes already at low dosages of an enzyme inhibiting drug, and less systematic effects once enzyme inhibition has occurred and dosages of the inhibitor are changed.

Changes in Brivaracetam serum concentrations may thus contribute to an increased efficacy observed with add-on treatment of CNB to a preixisting treatment with BRV. Similarly, potential clinical adverse effects may be related either to CNB or to BRV in this titration phase. Interestingly, despite of high serum concentrations of BRV throughout the titration phase of CNB only few substance-related adverse events were reported by patients. This may reflect the known high therapeutic index in SV2A ligands (Matagne et al., 2008). Nevertheless, there was a small number of patients reporting sleepiness without a direct correlation with elevated drug levels of BRV.

## Data Availability

The original contributions presented in the study are included in the article/supplementary material, further inquiries can be directed to the corresponding author.
